# LncRNA WAC-AS1 promotes osteosarcoma Metastasis and stemness by sponging miR-5047 to upregulate SOX2

**DOI:** 10.1186/s13062-023-00433-2

**Published:** 2023-11-14

**Authors:** Zhining Yang, Zhaoyong Liu, Weiqing Lu, Huancheng Guo, Jianzhou Chen, Ying Zhang

**Affiliations:** 1 Department of Radiotherapy, Cancer Hospital of Shantou University Medical College, No. 7 Raoping Road, Shantou, 515041 Guangdong PR China; 2 Department of Orthopaedics, First Affiliated Hospital of Shantou University Medical College, No.57 Changping Road, Shantou, 515041 Guangdong China

**Keywords:** Osteosarcoma, WAC-AS1, miR-5047, SOX2, Stemness

## Abstract

Cancer stemness and osteosarcoma (OS) malignant progression are closely associated. However, the molecular mechanisms underlying this association have not been fully demonstrated. Long noncoding RNAs (lncRNAs) are an intriguing class of widely prevalent endogenous RNAs involved in OS progression, the vast majority of which have not been characterized functionally. Here, we identified tumor promoter lncRNA WAC-AS1 to be highly expressed in OS tumors and associated with worse survival. Further analysis revealed that WAC-AS1 increased tumorsphere formation of OS cells and promoted metastasis, as confirmed by cell proliferation, transwell and wound healing assays. MiR-5047 was identified as a downstream target of WAC-AS1. Subsequently, based on bioinformatics analysis, RIP assay and luciferase reporter assay, SOX2 mRNA was verified as a target of miR-5047. WAC-AS1 enhanced OS cell proliferation and stemness via acting as a ceRNA by binding to miR-5047, thereby increasing SOX2 expression. In addition, SOX2 bound to the promoter region of *WAC-AS1* and promoted its transcription, thereby forming a positive feedback loop to regulate OS malignancy. Taken together, our findings show WAC-AS1 is a tumor promoter and a key regulator of OS cell stemness and metastasis via a miR-5047/SOX2 axis.

**Supplementary Information:**

The online version contains supplementary material available at 10.1186/s13062-023-00433-2.

## Introduction

Osteosarcoma (OS) is the most common primary bone tumor in children and adolescents, and often gains resistance to standard chemotherapies, highlighting the need for improved treatment regimens and identification of novel therapeutic targets [1]. Cancer stem cells (CSC) represent a sub-type of tumor cells and are attributed to tumor malignant progression [2]. Compared with cancer cells, the capacity of CSCs for self-renewal, differentiation and proliferation leads to worse outcomes, including chemo-resistance, cancer recurrence, and metastasis. Recent published work has linked CSC phenotypes to both drug resistance and tumorigenesis in OS [3]. The altered expression of genes, transcription factors, and signaling pathways, as well as non-coding RNAs, is thought to underlie enrichment of CSCs and increase cancer stemness [4]. Thus, identification of biomarkers and possible mechanisms will facilitate CSC-targeted elimination.

Long non-coding RNAs (lncRNAs) are non-coding, single-stranded molecules that regulate multiple molecular processes, including epigenetic, transcriptional, and post-transcriptional modulation of genes and proteins [5]. LncRNA plays pivotal roles in tumor cell growth, apoptosis, metastasis, and especially CSC maintenance [5, 6]. Among the lncRNAs in CSC studies, MALAT1, HOTAIR, and XIST have been the most studied [5]. However, more lncRNAs, such as NRAD1 in breast cancer and PVT1 in hepatocellular carcinoma, have been reported to modulate CSCs through fundamental signaling pathways and miRNA sponging [7, 8]. Mechanistically, lncRNA functions as a competing endogenous RNA (ceRNA) to regulate miRNA target genes, thereby affecting cell proliferation, apoptosis and CSC maintenance. For example, LOXL1-AS1 contributes to the development and stemness of gastric cancer via sponging miR-708-5p to upregulate USF1 [9], and FEZF1-AS1 functions as a tumor promoter in breast cancer by promoting cell stemness and tumorigenesis via the miR-30a/Nanog axis [10].

LncRNA WAC-antisense RNA1 (WAC-AS1) is the head-to-head antisense RNA of WAC (WW domain-containing adaptor with coiled-coil). The biological function of WAC-AS1 in cancer has been reported in a limited number of papers. Based on analysis of cohorts in TCGA, WAC-AS1 regulates immune responses, immune cell infiltration, and malignant properties in various types of cancers [11]. WAC-AS1 expression also correlates with the survival of ovarian cancer patients [12]. All this suggests that WAC-AS1 might be a useful prognostic marker in a wide range of cancers.

As a transcription factor, SOX2 recognizes and binds to the promoters of various target genes via its DNA-binding domain and trans-activates or trans-represses their expression, thus regulating various physiological processes [13]. Moreover, SOX2 plays important roles in the development of CSCs in a variety of tumors and maintains self-renewal and pluripotency in embryonic stem cells [14]. In the case of OS patients, elevated SOX2 expression is positively correlated with drug resistance and poor survival [15]. Therefore, targeting SOX2 appears to be a very attractive therapeutic avenue for OS treatment.

In this study, we show that lncRNA WAC-AS1 functions as a tumor promoter in OS by maintaining stemness through inducing SOX2 expression by serving as a ceRNA for miR-5047.

## Materials and methods

### Clinical samples and cell lines

This study used formalin-fixed and paraffin-embedded OS patient tissues (n = 72) and normal tissues (n = 6), as well as fresh primary OS tissues (n = 20) and normal tissues (n = 5) that were collected from the Sun Yat-sen University Cancer Center, First Affiliated Hospital of Shantou University Medical College and First Affiliated Hospital of Zhejiang University Medical College from 2010 to 2023. None of the patients had received preoperative radiotherapy or chemotherapy. This study was approved by the ethical review committees of the Shantou University Medical College Cancer Center. All participants involved in our study provided written informed consent.

OS cell lines (MG63, U2OS), obtained from Cell Bank of the Chinese Academy of Science (Shanghai, China), were cultured in Dulbecco’s modified Eagle’s medium (DMEM) supplemented with 10% fetal bovine serum (FBS, Gibco, Shanghai, China), at 37^o^C in a humidified incubator containing 5% CO _2_.

### Drugs, siRNA, plasmid transfection and stable cell lines

Paclitaxel and cisplatin were purchased from MCE (Shanghai, China). For knockdown, short interfering RNAs against SOX2 (si-SOX2), miR-5047 and control mimics and inhibitors were purchased from the GenePharma Company (Shanghai, China). For gene transfection, WAC-AS1, pcDNA3.1-SOX2 and the negative control vectors (NC) were purchased from the Vigene Company (Jiangsu, China). jetPRIME (Polyplus Transfection, USA) was used to transfect the above-mentioned plasmids or oligonucleotides. Transfection was performed according to the user’s manual. For stable cell lines, WAC-AS1 shRNA and control shRNA were separately inserted into the pGLV3/H1/GFP/Puro vector. Wildtype and mutant WAC-AS1 sequences were cloned into the lentiviral expression vector pCDGEF1/GFP/Puro. OS cells were infected by the lentivirus following the user’s instructions. To isolate stably expressing cells, the infected cells were selected in 1 µg/ml puromycin (Gibco, USA) for two weeks.

### RNA sequencing and qRT-PCR

RNA sequencing was performed by Gene Denovo Biotechnology Co. (Guangzhou, China) according to the manufacturer’s protocol. Briefly, total RNA was extracted using Trizol reagent (Invitrogen, Carlsbad, CA,USA) The enriched mRNAs and ncRNAs were fragmented into short fragments and reverse transcribed into cDNA with random primers. After purification and ligation to Illumina sequencing adapters, the second-strand cDNA was digested and selected by agarose gel electrophoresis, PCR amplified, and sequenced using an Illumina HiSeqTM 4000. The RNA-seq data is available online: SRA accession: PRJNA640969.

Cytoplasmic and nuclear RNA extraction was performed using a FastPure Cytoplasmic & Nuclear RNA purification kit (ECOTOP Scientific, Guangzhou). Total RNAs were extracted using an RNA extraction kit purchased from the Vazyme Company (Nanjing, China). The cDNA was reverse-transcribed using a cDNA Synthesis Kit (Vazyme). Then, SYBR Green Master Mix (Vazyme) was used to perform qRT-PCR in a Bio-Rad 7500 Fast RT-PCR System. β-actin and U6 served as controls. The primer sequences are listed in **Table ****S1**.

### Cell proliferation, colony formation and migration assays

A Cell Counting Kit-8 (CCK-8) (Solarbio, China) was utilized to measure cell proliferation as previously described [16]. For colony formation, 500 cells were seeded in a 6-well plate and stained after 14 days. Colonies with > 30 cells were scored. Transwell and wound healing assays were used to examine cell migration as previously described [16].

### Tumorsphere formation assay

One thousand cells were seeded in ultralow attachment six well plates (Corning) and cultured for 1 week in DMEM/F12 medium (Invitrogen, Shanghai, China) supplemented with B27 (1:50, Gibco), 20 ng/ml bFGF (Sigma, Shanghai, China), and 20 ng/ml EGF (Sigma). Pictures were taken using a bright-field microscope and the number of the spheres was counted.

### Flow cytometry analysis

Cell apoptosis was examined using an Annexin V-FITC/PI Double Staining Kit (Beyotime, China) according to the user’s manual. To detect the OS stem cell subpopulations, APC-anti-CD133 antibody was used. A total of 1 × 10^6^ cells were incubated with antibodies in the dark at 4 °C for 30 min. After washing, the cells were re-suspended in 500 µl of PBS and analyzed using a flow cytometer(C6, Japan).

### Fluorescence in situ hybridization (FISH), ISH and immunohistochemistry (IHC)

FISH staining was performed according to the user’s manual. MG63 and U2OS cells, grown on slides, were fixed with 4% (v/v) formaldehyde, and then dehydrated for overnight hybridization with a WAC-AS1 probe. After counterstaining with DAPI, slides were examined under a fluorescence microscope. For ISH, WAC-AS1 probes were synthesized by Boster (Wuhan, China). WAC-AS1 expression was also examined in formalin-fixed, paraffin-embedded (FFPE) samples using procedures outlined in the user’s manual. IHC was performed as described previously [16]. The scores for ISH and IHC in OS samples were quantified by two pathologists, and the average score of each tissue was obtained for statistical analysis.

### Western blot analysis

Total protein from cells and tissues was extracted by RIPA lysis buffer (Beyotime) containing 1% protease inhibitors (Invitrogen). Western blotting was performed as previously described [16]. The primary antibodies were anti-SOX2, Nanog, OCT4, PARP, CD133, Bax, Bcl-2, β-actin and GAPDH (all CST, 1:1000).

### RNA immunoprecipitation (RIP) assay

A RIP Kit (Bersinbio, Guangzhou, China) was utilized to determine the binding between WAC-AS1, miR-5047 and AGO2 protein according to the manufacturer’s protocol. In brief, OS cells were washed with PBS and resuspended using RIP lysis buffer containing a proteasome inhibitor. Antibody against AGO2 or IgG was incubated with cell lysates overnight. Then RNA-protein complexes were incubated with protein A/G magnetic beads. After proteinase K digestion, the immunoprecipitated RNAs were purified and subjected to qRT-PCR.

### Chromatin immunoprecipitation (ChIP) assay

A ChIP Kit (Bersinbio) was used, to determine the binding between SOX2 protein and the predicted *WAC-AS1* promoter region, according to the manufacturer’s instructions. In brief, cells were crosslinked in 1% formaldehyde and quenched with glycine. Then 200–600 bp fragments of cross-linked chromatin were obtained by sonication, and then incubated with SOX2 or IgG antibody overnight. Finally, DNA was isolated and quantitative PCR was used to examine the relative enrichment of the *WAC-AS1* promoter region.

### Dual luciferase reporter assay

WAC-AS1 cDNA, either wild-type (WAC-AS1 WT) or containing a mutant miR-5047 binding site (WAC-AS1 Mut), were cloned into the pmirGLO vector (Vigene). Then, the indicated plasmids were co-transfected with miR-5047 mimics or control into OS cells. After co-transfection for 48 h, a dual Luciferase Reporter Assay System (Promega, MD, USA) was used to detect relative luciferase activity according to the manufacturer’s instructions.

### RNA pulldown assay

To capture proteins capable of binding to WAC-AS1, a desthiobiotin-labeled WAC-AS1 was designed. An RNA Pull-Down Kit (Bersinbio) was used according to the manufacturer’s instructions. The biotin–labeled WAC-AS1 was incubated with OS cell protein extracts, then pulled down with magnetic beads and eluted. Anti-AGO2 was detected by western blotting, and miR-5074 was detected by qRT-PCR.

### MiRNA target prediction

MiRNAs that potentially bound to the 3’ untranslated region (3’UTR) of an mRNA or lncRNA were predicted using websites, including ENCORI (https://starbase.sysu.edu.cn/), miRDB (http://www.mirdb.org/) and Targetscan (http://www.targetscan.org/vert_72/). MiRNAs potentially sponged by WAC-AS1 were predicted via ENCORI and miRDB. Overlapping miRNAs were chosen as miRNA candidates sponged by WAC-AS1.

### Animal studies

U2OS cells transduced with WAC-AS1 or NC lentivirus were subcutaneously injected into the backs of BABL/c nude mice (n = 6). The tumor volume of each mouse was calculated every 7 days according to the formula: length× width^2^ × 0.5. Four weeks after cell injection, mice were euthanized and the xenografted tumors were collected and weighed. Then, the growth curves of tumors of the two groups were drawn according to the tumor volume. For metastasis, stable WAC-AS1 expression or control OS cells were injected into mice through tail vein (n = 5). After 5 weeks, euthanasia was performed, and the numbers of lung metastatic nodules were counted under the microscope.

### Statistical analysis

Data analysis was conducted with SPSS 19.0 or GraphPad prism 8.0 software. The results are presented as mean ± SD after three independent trials. Student’s t test or one-way analysis of variance (ANOVA) was implemented to compare statistical differences between two groups or among multiple groups. Associations between different target expressions in OS patients were analyzed using Pearson’s correlation analysis. A P < 0.05 was considered to indicate statistical significance.

## Results

### WAC-AS1 expression is upregulated in OS tissues

**Fig. 1:**
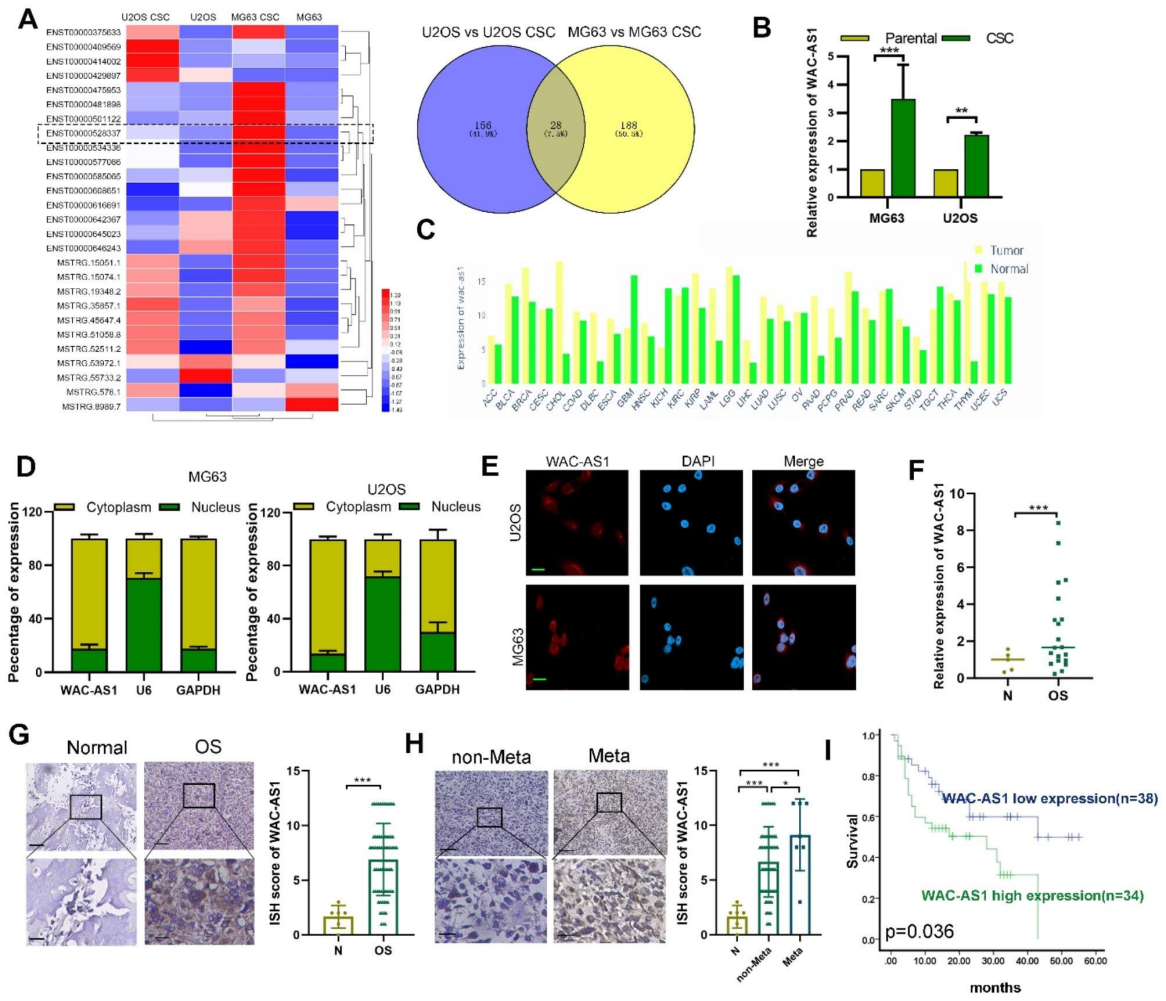
**Expression of WAC-AS1 in OS and its clinical significance. A.** Heatmap of differentially-expressed lncRNAs in cancer stem cells (CSCs) and control cells is shown based on RNA-seq data. Venn diagram displaying 28 differentially-expressed lncRNAs in CSCs vs. parental OS cells for both MG62 and U2OS cell lines. **B.** Expression of WAC-AS1 was detected in parental MG63 and U2OS osteosarcoma cells and CSCs. **C**. WAC-AS1 expression was higher in cancer tissues than normal tissues, based on TCGA analysis. **D**. Expression of WAC-AS1 in the cytoplasm and nucleus of OS cells as determined by qRT-PCR. **E**. Location of WAC-AS1 was detected by fluorescence in situ hybridization (FISH) (scale bar: 20 μm). **F**. Expression of WAC-AS1 was detected by qRT-PCR in OS tissues (n = 20) and normal tissues (n = 5). **G.** Expression of WAC-AS1 was detected by ISH in 72 OS cases and 6 normal tissues (scale bar: 100 μm, 25 μm). **H**. Expression of WAC-AS1 was detected by ISH in tumors from 63 OS non-metastasis cases and 9 metastasis cases (scale bar: 100 μm, 25 μm). **I**. Survival curve of OS patients with high and low WAC-AS1 expression is shown. Error bars represent three independent experiments, *, p < 0.05, **p < 0.01, *** p < 0.001

To identify lncRNAs involved in OS stemness, we conducted RNA sequencing of tumorspheres and parental MG63 and U2OS cells. LncRNAs with differential expression (fold change ≥ 1 and p < 0.05) between spheres and parental groups were identified. As shown in Figs. 1A and 28 lncRNAs were differentially expressed in spheres compared with parental OS cells in both cell lines. We then further examined the expression of the 5 most increased lncRNAs and identified a novel lncRNA (ENST00000528337), termed WAC-AS1, located on human chromosome 10p12.1, that was significantly upregulated in CSCs compared with parental cells(Fig. 1B and S1). In TCGA, WAC-AS1 was upregulated in most types of cancer tissues compared with normal tissues (Fig. 1C). Furthermore, cellular fractionation assays and FISH results showed that WAC-AS1 was mainly localized in the cytoplasm of OS cells (Fig. 1D, E).

Then, we explored the expression and clinical significance of WAC-AS1 in OS. First, we detected the expression of WAC-AS1 in OS (n = 20) and normal tissues (n = 5) by qRT-PCR and found WAC-AS1 expression to be higher in OS tissues than in normal tissues (Fig. 1F). A similar result was found, using ISH, in a cohort of OS clinical samples (n = 72) and normal tissues (n = 6) (Fig. 1G). As shown in Fig. 1H, OS tissues with metastasis had the highest expression compared with non-metastatic tumors and normal tissues. WAC-AS1 expression correlated with OS patient gender and lung metastasis (Table 1), Kaplan–Meier survival analysis revealed that high level WAC-AS1 expression was related to poor patient prognosis (Fig. 1I). However, it was not verified as a risk factor for overall patient survival in multivariable analysis (**Table ****S2**).

**Table 1:** Correlation between WAC-AS1 expression and OS clinical parameters

Parameters		WAC-AS1 expression		Number	*P*
		Low (n = 34)	High (n = 38)		
**Age**	> 25	11	18	29	0.23
	≤ 25	23	20	43	
**Gender**	Male	18	30	48	0.025*
	Female	16	8	24	
**Location**	Tibia	9	12	21	0.88
	Fibula	0	3	3	
	Thighbone	17	9	26	
	Other	8	14	22	
**Lung Metastasis**	Yes	1	8	9	0.03*
	No	33	30	63	
**Invasion**	Yes	3	6	9	0.485
	No	31	32	63	
**Lymph node metastasis**	Yes	2	2	4	1
	No	32	36	68	
**Tumor size**	> 4 cm	19	23	42	0.81
	< 4 cm	15	15	30	

### WAC-AS1 promotes cell proliferation, migration and stemness of OS cells

**Fig. 2:**
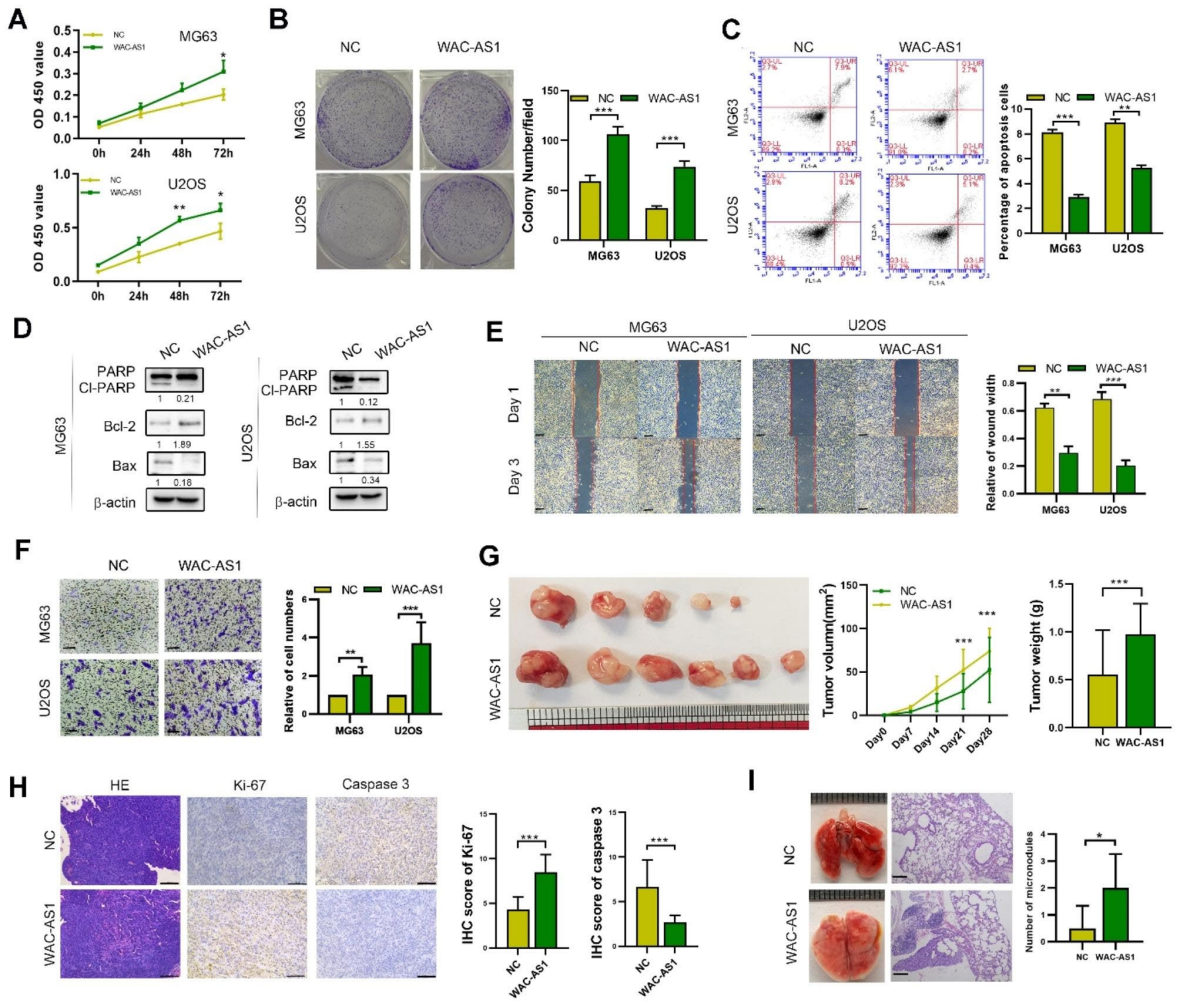
**Determination of WAC-AS1 function in OS*in vivo and in vitro***. **A**. CCK8 assay measuring the proliferation of WAC-AS1-transfected or control (NC) OS cells. **B**. Colony formation by WAC-AS1-transfected or control OS cells. **C.** Flow cytometry detecting apoptosis of WAC-AS1-transfected or control OS cells. **D**.PARP and cleaved PARP (cl-PARP), Bcl-2 and Bax were detected by western blotting in WAC-AS1 and control OS cells. **E**. Wound healing assay showing the migration of WAC-AS1-transfected or control OS cells (scale bar, 100 μm). **F.** Transwell assay showing the migration of WAC-AS1-transfected or control OS cells (scale bar, 50 μm). **G**. Representative images of xenograft tumors (n = 6) resulting from WAC-AS1-overexpressing and control cells. Tumor growth curves and tumor weight are shown. **H.** H&E staining and IHC staining of caspase-3 and Ki67 in control and WAC-AS1-overexpressing tumors (scale bar, 100 μm). **I.** Lung metastases (n = 5) following tail vein injection of WAC-AS1-overexpressing and control cells (scale bar, 100 μm). Error bars represent three independent experiments, *, p < 0.05, **p < 0.01, *** p < 0.001

We transfected or knocked down WAC-AS1 in MG63 and U2OS cells, and validated the knockdown levels by qRT-PCR (**Fig. ****S2****A**). CCK8 assay showed WAC-AS1 silencing reduced, while WAC-AS1 overexpression enhanced OS cell proliferation in both OS cell lines (Fig. 2A **and Fig. ****S2****B**). Similarly, the colony forming ability of OS cells was increased by WAC-AS1 transfection compared with control (Fig. 2B). In addition, the percentage of cell apoptosis, detected by flow cytometry, and protein expression of PARP were decreased by WAC-AS1 transfection (Fig. 2C, D). WAC-AS1 promoted, while WAC-AS1 knockdown reduced OS cell migration, as determined by transwell and wound healing assays (Fig. 2E, F and S2C). To further confirm our *in-vitro* results, a stable WAC-AS1-overexpressing OS cell line xenograft model was established to evaluate the influence of WAC-AS1 on xenograft tumor volume and metastasis. WAC-AS1 overexpression facilitated xenograft tumor growth and metastasis in vivo (Fig. 2G-I). Also, WAC-AS1 overexpression reduced caspase-3 and enhanced Ki-67 expression (Fig. 2I). Thus, we draw the conclusion that WAC-AS1 promotes cell proliferation and metastasis in OS cells.

WAC-AS1 overexpression increased number of CD133 + OS cells and tumorspheres in the U2OS and MG63 cell lines, whereas knock down of WAC-AS1 decreased the percentage of CD133 + OS cells (**Fig. ****S2****D,E**). WAC-AS1 also enhanced the expression of stemness biomarkers (CD133, SOX2 and Nanog), as judged by western blotting and qRT-PCR (**Fig. ****S2****F**). CSCs, a subset of cancer cells, drives tumor progression, metastasis and drug resistance [17]. To further verify the role of WAC-AS1 on drug resistance, the effects of WAC-AS1 on response to chemotherapeutic drugs (paclitaxel and cisplatin) were investigated. WAC-AS1 increased the IC50 of these drugs, while knockdown of WAC-AS1 decreased the IC50, indicating WAC-AS1 reduced OS sensitivity to chemotherapeutic drugs (Fig. 2G). These results indicate that WAC-AS1 mediates OS stemness and chemo-resistance.

### WAC-AS1 upregulates SOX2 expression by acting as a ceRNA for miR-5047

**Fig. 3:**
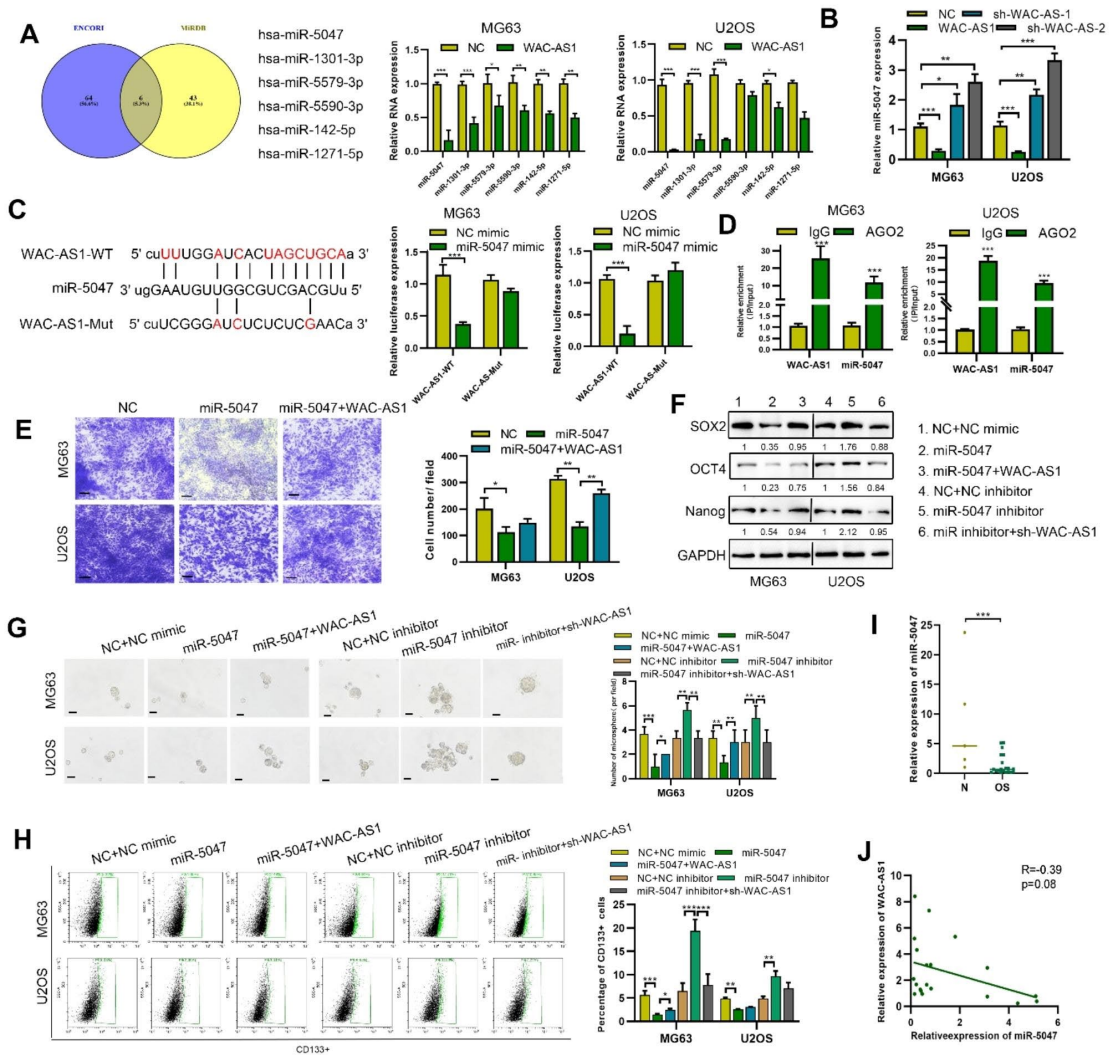
**MiR-5047 is a target of WAC-AS1**. **A**. Prediction of target miRNAs potentially binding to WAC-AS1 was performed using ENCORI and miRDB. Expression of miRNAs was determined in control and WAC-AS1-transfected OS cells. **B**. Expression of miR-5047 was detected after transfecting WAC-AS1 or sh-WAC-AS1. **C**. Wild-type and mutant WAC-AS1 binding sites in miR-5047. Luciferase reporter assay detecting the binding of miR-5047 to WAC-AS1 in OS cells. **D**. RIP using anti-AGO2 antibody was performed to detect WAC-AS1 and miR-5047. **E**. Transwell assay performed to detect the migtaion of WAC-AS1- or miR-5047-transfected or control OS cells (scale bar, 50 μm). **F**. Western blotting to determine stemness-related proteins in control and indicated transfected cells. **G.** Changes in tumorsphere-forming ability of control and indicated transfected OS cells (scale bar, 50 μm). **H.** Percentage of CD133 + cells in WAC-AS1-overexpressing, SOX2-transfected or WAC-AS1-knockdown and control OS cells was determined by flow cytometry. **I**. Expression of miR-5047 was measured by qRT-PCR in OS tissues and normal tissues. **J.** Correlation between WAC-AS1 and miR-5047 expression in OS. Error bars represent three independent experiments, *p < 0.05, **p < 0.01, *** p < 0.001

Then we explored the mechanism underlying WAC-AS1-regulated OS malignant procession and stemness. Cytoplasmic lncRNA can work as a molecular sponge for miRNAs. Thus, we used bioinformatic tools (MiRDB and ENCORI) to predict miRNAs potentially targeted by WAC-AS1. This resulted in six miRNAs that were common to both prediction tools. Then, we used qRT-PCR to analyze the expression of these miRNAs in WAC-AS1 overexpressing OS cells and found miR-5047 to be the most downregulated (Fig. 3A). The expression of miR-5047 was upregulated in WAC-AS1-knockdown OS cells and downregulated in WAC-AS1-overexpressing cells (Fig. 3B). Therefore, miR-5047 was selected as the top candidate miRNA for targeting WAC-AS1 in this study.

The putative binding site between miR-5047 and WAC-AS1 is shown in Fig. 3C. In dual luciferase reporter assays, there was a significant reduction in relative luciferase activity following co-transfection of miR-5047 mimics and a WAC-AS1-WT reporter vector transfection, compared with co-transfection with a mutated reporter, thereby confirming miR-5047 to be a direct target of WAC-AS1 in OS cells. RIP assays further indicated that both WAC-AS1 and miR-5047 were enriched in the AGO2 pulldown group but not the IgG group in MG63 and U2OS cells (Fig. 3D). These results indicate that WAC-AS1 acts as a molecular sponge for miR-5047. MiR-5047 reduced the tumor-promoting effects of WAC-AS1 on OS cell migration, proliferation apoptosis, colony formation and stemness (Fig. 3E-H and S3A-E). Then we detected the expression of miR-5047 by qRT-PCR in OS and showed that miR-5047 was clearly decreased in OS compared with normal tissues (Fig. 3I). Analysis of the correlation, between expression of WAC-AS1 and miR-5047, in OS patient samples, showed a tendency for negative correlation that was not significant (Fig. 3J). Nevertheless, these results indicate that the oncogenic role of WAC-AS1 is mediated through the regulation of miR-5047 expression.

### MiR-5047 targets SOX2 to negatively regulate SOX2 expression

**Fig. 4:**
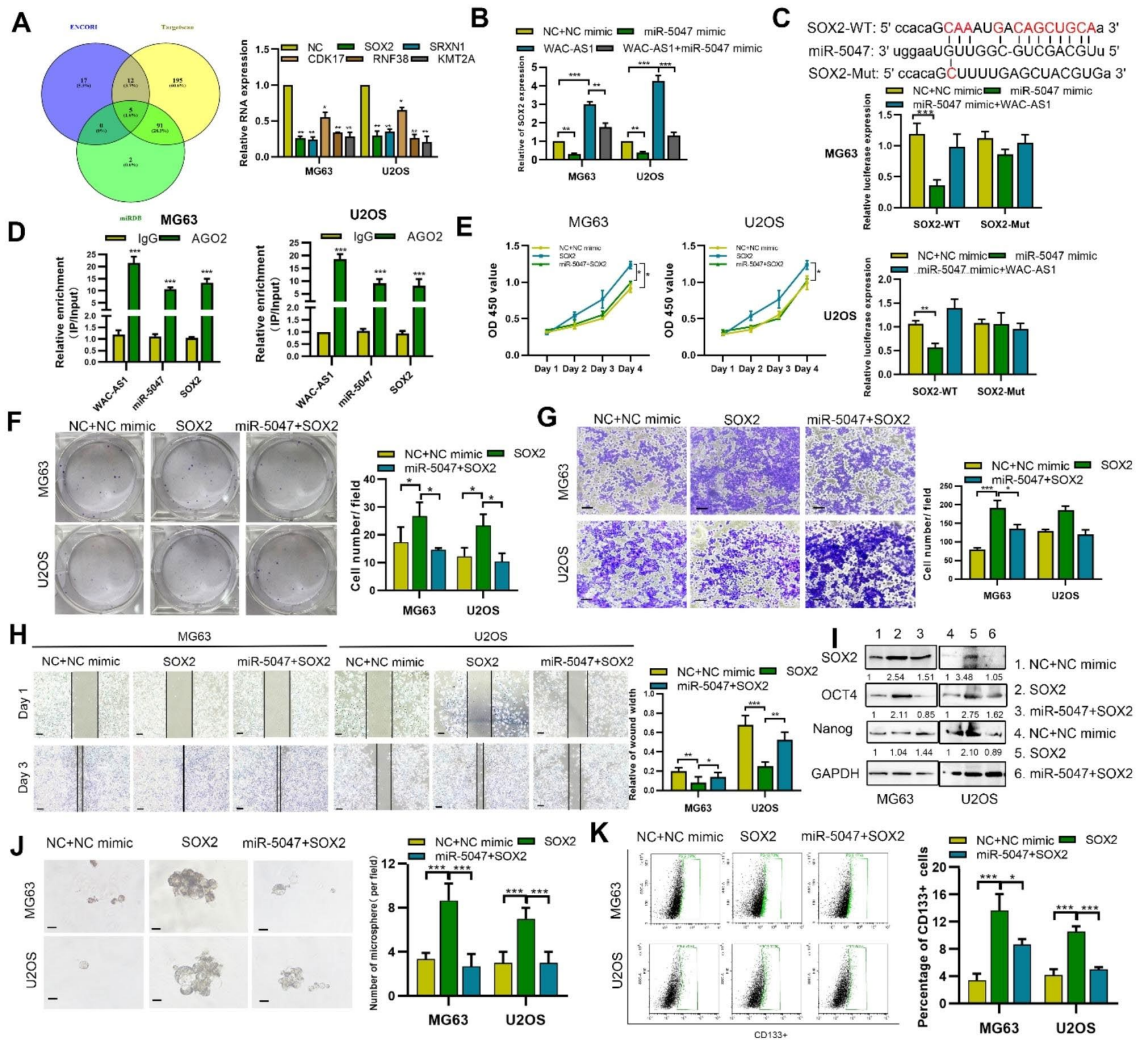
**MiR-5047 regulates OS stemness and malignancy by targeting SOX2. A.** ENCORI, TargetScan and miRDB prediction of potential targets binding to miR-5047. Expression of indicated target genes was detected after transfecting the indicated miR-5047 mimics. **B**. Expression of SOX2 was detected in WAC-AS1-, miR-5047- or control-transfected cells. **C.** Hypothetical wild-type and mutant binding sites in miR-5047 for SOX2 3’UTR. Luciferase reporter assay detecting the binding of miR-5047 to SOX2 in OS cells. **D**. RIP with anti-AGO2 antibody was performed to detect WAC-AS1, miR-5047 and SOX2 mRNA. **E**. CCK8 assay showing the proliferation of OS cells following transfection of SOX2, miR-5047 + SOX2 or control. **F.** Colony formation of OS cells following transfection of SOX2, miR-5047 + SOX2 or control. **G**. Transwell assay showing the migration of miR-5047- or SOX2-transfected or control OS cells (scale bar, 50 μm). **H.** Wound healing assay the migration of SOX2 transfected, miR-5047-transfected or control OS cells (scale bar, 100 μm). **I**. Western blots showing stemness-related proteins in miR-5047-transfected, miR-5047 + SOX2-transfected or control cells. **J.** Changes in tumorsphere-forming ability of SOX2-transfected, miR-5047-transfected or control OS cells (scale bar, 50 μm). **K.** Percentage of CD133 + cells in populations of miR-5047-transfected, miR-5047 + SOX2-transfected or control OS cells was determined by flow cytometry. Error bars represent three independent experiments, *p < 0.05, **p < 0.01, *** p < 0.001

Using the ENCORI database, Targetscan and miRDB were used to identify miR-5047 targets (Fig. 4A). Among these targets, SOX2 was one of significantly downregulated in miR-5047-mimic transfected OS cells, whereas WAC-AS1 enhanced expression of SOX2 (Fig. 4B **and Fig. ****S4****A**). Furthermore, luciferase reporter assays showed that miR-5047 mimics caused a reduction in luciferase activity of a reporter gene bearing the wildtype SOX2 3’ UTR sequence, but not a mutant SOX2 3’UTR sequence (Fig. 4C). Importantly, co-transfection of WAC-AS1 reversed the inhibitory effect of miR-5047 on luciferase activity of the wildtype SOX2 3’UTR plasmid, indicating the regulatory role of WAC-AS1 on miR-5047 for SOX2 expression (Fig. 4C). Furthermore, WAC-AS1, miR-5047 and SOX2 mRNA were abundantly enriched in the AGO2 group (Fig. 4D).

As shown in Fig. 4E-H and S4B, transfection of SOX2 reversed the effects of miR-5047 overexpression on OS cell proliferation, apoptosis, colony formation and migration. Stemness induced by SOX2 overexpression in OS cells was also abolished by a miR-5047 mimic (Fig. 4I-K and S4C). Taken together, our data demonstrate that WAC-AS1 up-regulates SOX2 through sponging miR-5047.

### A WAC-AS1/SOX2 feedback loop promotes OS malignant progression and stemness

**Fig. 5:**
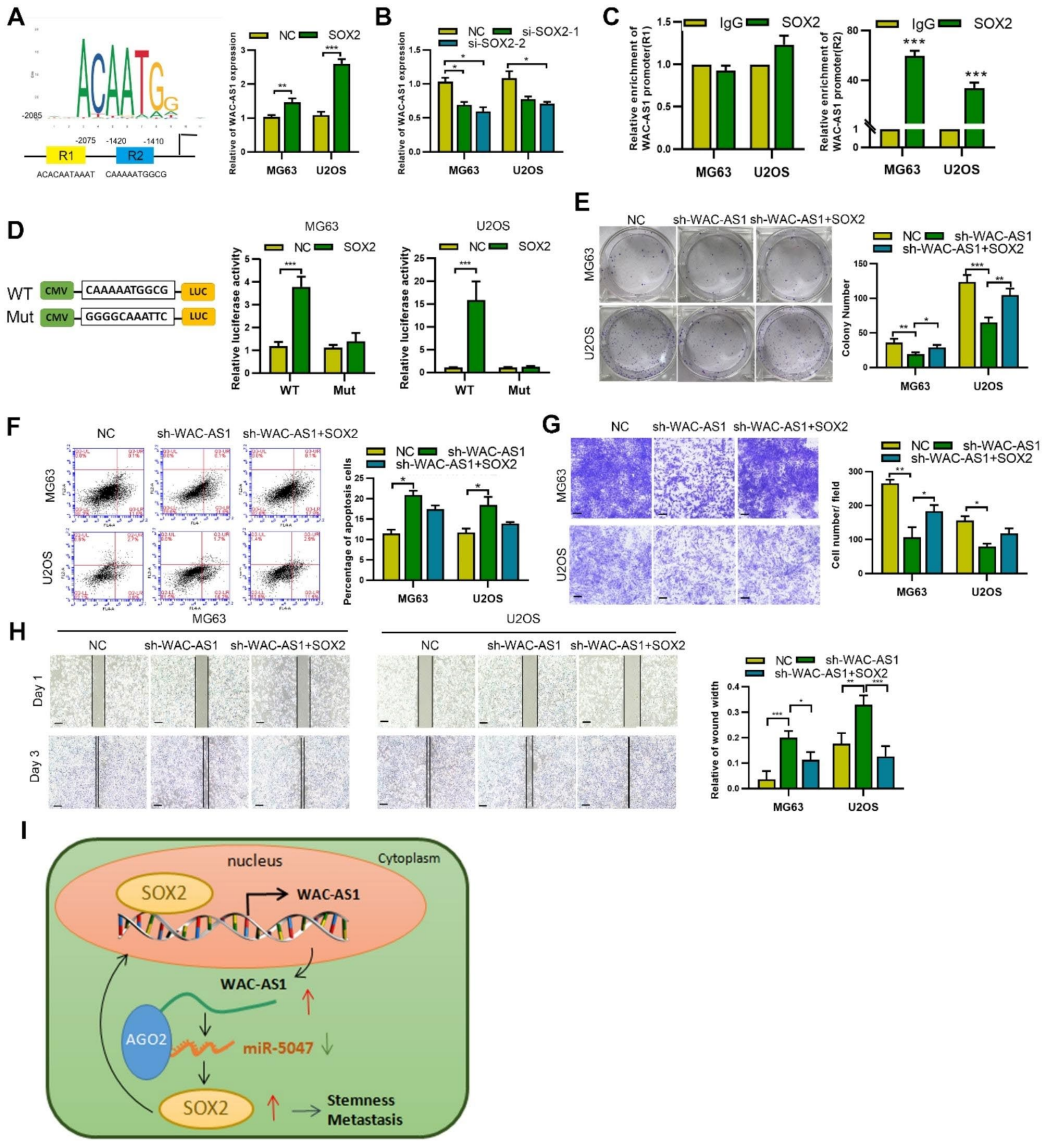
** A WAC-AS1/SOX2 feedback loop enhances OS malignancy. A.** JASPAR was utilized to predict the possible binding sites of transcription factor SOX2 in the *WAC-AS1* promoter region. **B**. WAC-AS1 expression was determined, by qRT-PCR, after SOX2 transfection or knockdown on in OS cells. **C**. ChIP was used to examine the binding of SOX2 with the *WAC-AS1* promoter at two predicted sites (R1 and R2). **D**. Schematic diagram of luciferase reporter vectors containing a wild-type or mutant R2 sequence in the *WAC-AS1* promoter. Dual luciferase reporter assay was performed in OS cells transfected with the wild-type or mutant reporter. **E.** Colony formation of sh-WAC-AS1-transfected, SOX2-transfected or control OS cells. **F.** Flow cytometry detecting apoptosis of sh-WAC-AS1-transfected, SOX2-transfected or control OS cells. **G.** Transwell assay showing the migration of sh-WAC-AS1-transfected, SOX2-transfected or control OS cells(scale bar, 50 μm). **H.** Wound healing assay showing the migration of sh-WAC-AS1-transfected, SOX2-transfected or control OS cells(scale bar, 100 μm). **I**. Schematic diagram of this project. Error bars represent three independent experiments, *p < 0.05, **p < 0.01, *** p < 0.001

Since SOX2 is a key transcription factor in OS and its expression level positively correlated with WAC-AS1 (**Fig. ****S5****A**), we speculated that SOX2 might bind to the promoter region of *WAC-AS1*. First, we used JASPAR (http://jaspar.genereg.net/) to predict potential binding sites of SOX2 in the *WAC-AS1* promoter region and obtained two putative binding regions (Fig. 5A). SOX2 depletion reduced expression of WAC-AS1, whereas SOX2 transfection increased the expression (Fig. 5B). Second, we performed a ChIP assay and found that SOX2 protein directly bound to the predicted R2 binding site (− 1420 to − 1410) of the *WAC-AS1* promoter (Fig. 5C). Moreover, SOX2 remarkably enhanced the activity of the *WAC-AS1* promoter, as judged by a luciferase reporter assay. However, luciferase activity was reduced when the SOX2 binding sites in the *WAC-AS1* promoter were mutated (Fig. 5D). These results indicate that SOX2 can bind to the promoter of WAC-AS1.

SOX2 plays a critical role in maintaining the self-renewal and stemness of CSCs [11], thus we further hypothesized that WAC-AS1 promotes malignancy and stemness in OS via SOX2. We transfected the SOX2 plasmid in control or WAC-AS1-knockdown OS cells and verified the transfection efficiency by qRT-PCR and western blotting (**Fig. ****S5****B, C**). SOX2 overexpression rescued the suppressive effects of WAC-AS1 silencing on OS cell proliferation and migration, as shown by CCK-8 and colony formation assays, as well as transwell and wound healing assays (Fig. 5E-H and S5D), and flow cytometry revealed that SOX2 overexpression reversed the increase in apoptosis caused by WAC-AS1 knockdown (Fig. 5F). Compared with the control group, the protein levels of OCT4 and Nanog were decreased after WAC-AS1 depletion, and SOX2 overexpression reversed the WAC-AS1 depletion-mediated decrease in levels of these proteins (**Fig. ****S5****C**). Moreover, SOX2 overexpression also rescued the inhibitory effects of WAC-AS1 knockdown on the chemoresistance and number of CD133 + OS cells (**Fig. ****S5****E-G**). In summary, these findings indicate that WAC-AS1 and SOX2 form a positive feedback loop to facilitate OS cell proliferation and metastasis.

## Discussion

CSCs are key regulators of cancer metastasis, chemo-resistance and relapse [17]. Accumulating evidence shows that lncRNAs play pivotal roles in CSCs [18]. Here, we identified a CSC-related lncRNA WAC-AS1 that is highly expressed in OS, and correlates with poor survival. Furthermore, we demonstrate that WAC-AS1 promotes tumorigenesis and stemness by sponging miR-5047 to upregulate SOX2 expression and ultimately increase the self-renewal and chemo-resistance of OS cells by regulating a miR-5047/SOX2 axis (Fig. 5I).

The function of WAC-AS1 has been demonstrated in a limited number of cancer types. In hepatocellular carcinoma, WAC-AS1 correlates with poor prognosis and promotes glycolysis and cell proliferation by regulating miR-320d/ARPP19 [19]. In glioma, WAC-AS1 has been identified as one of the prognostic ferroptosis-related lncRNAs and correlates with immune landscape and radiotherapy response [20]. However, the expression and function of WAC-AS1 in OS has not yet been demonstrated. To investigate the function of WAC-AS1 in OS, we performed a series of functional experiments and found that WAC-AS1 increases OS cell proliferation and migration, and inhibits apoptosis. That WAC-AS1 acts as an oncogene in OS is further supported by in vivo experiments showing that WAC-AS1 promotes OS cell proliferation and metastasis. Moreover, WAC-AS1 increases tumorsphere formation and the expression of SOX2, a master regulator of cancer stemness and CSC self-renewal, while decreasing the chemo-sensitivity of OS cells, indicating that WAC-AS1 regulates the stemness of OS.

Accumulating evidence indicates that lncRNAs exert their effects mainly through three mechanisms: cis regulation of parental gene expression, miRNA sponging, and binding to RNA-binding proteins (RBPs) [6]. For example, lncRNA SNHG1 promotes OS progression through mediating S100A6 expression by competitively sponging miR-493-5p [21]. To explore the possible role of WAC-AS1 in OS, we first determined that the location of WAC-AS1 is mainly cytoplasmic. Thus, we suspected that WAC-AS1 may function as a miRNA sponge. Using RIP, we found that AGO2, a miRNA-mediated gene silencing RBP, can bind to WAC-AS1. By qRT-PCR, miR-5047 was identified as a downstream target miRNA of WAC-AS1 and that miR-5047 is the most downregulated miRNA after WAC-AS1 transfection among the six miRNAs predicted by bioinformatics analysis. Subsequently, luciferase reporter, RIP and FISH assays were conducted to verify the interaction of WAC-AS1 and miR-5047. However, the role of miR-5047 in cancer has not been elucidated. In head and neck squamous cell carcinoma and cervical cancer, miR-5047 acts as a tumor suppressor in cancer since its expression is higher in normal tissues compared with tumors [22]. Overexpressing miR-5047 inhibits cervical carcinoma cell metastasis by downregulating VEGFA expression [23]. Our functional experiments demonstrate that miR-5047 inhibition can restore the attenuated stemness and proliferation resulting from WAC-AS1-knockdown in OS cells.

Furthermore, we show SOX2, one of the most well-known cancer stemness biomarkers, is a downstream target of miR-5047 and that SOX2 mRNA can directly interact with miR-5047 in OS cells, suggesting that WAC-AS1 may regulate the expression of SOX2 via competitively binding with miR-5047. Additionally, SOX2 levels are upregulated in both WAC-AS1-overexpressing and miR-5047 knockdown OS cells. Overexpression of SOX2 could rescue the attenuated tumor phenotype resulting from WAC-AS1 knockdown and miR-5047 mimic treatment. SOX2 has been previously reported to function as an oncogene in various cancers, including breast cancer, prostate cancer, renal cell carcinoma and thyroid cancer [24]. Moreover, SOX2 has been shown to be essential for the survival and proliferation of OS cells in a mouse tumor model [15]. We extend these studies by showing that not only does WAC-AS1 enhance SOX2 expression by serving as a miR-507 sponge, but also that SOX2 can serve as a transcription factor to upregulate WAC-AS1 expression by binding to the *WAC-AS1* promoter. Thus, WAC-AS1/SOX2 forms a positive feedback loop to regulate OS tumorigenesis and chemoresistance.

In conclusion, our study identifies WAC-AS1 as a novel oncogenic lncRNA in OS by regulating a WAC-AS1/miR-5047/SOX2 axis. WAC-AS1 promotes OS cell proliferation, stemness and migration by upregulating SOX2 through sponging miR-5047. Furthermore, SOX2 itself increases the transcription of WAC-AS1. Taken together, we reveal a novel role of WAC-AS1 in OS proliferation and metastasis by regulating a miR-5047/SOX2 axis in a positive feed loop, which might be a potential target for the treatment of OS.

### Electronic supplementary material

Below is the link to the electronic supplementary material.

Supplementary Material 1

Supplementary Material 2

Supplementary Material 3

Supplementary Material 4

Supplementary Material 5

Supplementary Material 6

Supplementary Material 7

## Data Availability

All data generated or analyzed in this study are included in this article, and are available from the corresponding author on request.

## Abbreviations

CCK-8: Cell Counting Kit-8
CDDP: Cisplatin
ChIP: Chromatin immunoprecipitation
ceRNA: Endogenous RNA
CSC: Cancer stem cell
DAPI: 4’,6-diamidino-2-phenylindole
DMEM: Dulbecco’ s modified Eagle’ s medium
EMT: Epithelial-mesenchymal transition
FBS: Fetal bovine serum
FISH: Fluorescence in situ hybridization
GAPDH: Glyceraldehyde-3-phosphate dehydrogenase
GO: Gene Ontology
IHC: Immunohistochemistry
IP: Immunoprecipitation
LncRNA: Long noncoding RNA
NC: Negative control
OS: Osteosarcoma
PBS: Phosphate-buffered saline
PTX: Paclitaxel
qRT-PCR: Quantitative reverse transcription polymerase chain reaction
TAD: Transcription activation domain
