# Supplementary figures and images for: LncRNA WAC-AS1 promotes osteosarcoma Metastasis and stemness by sponging miR-5047 to upregulate SOX2
Source: Biol Direct. 2023 Nov 14;18:74. doi: 10.1186/s13062-023-00433-2 (PMC10644615; doi:10.1186/s13062-023-00433-2)

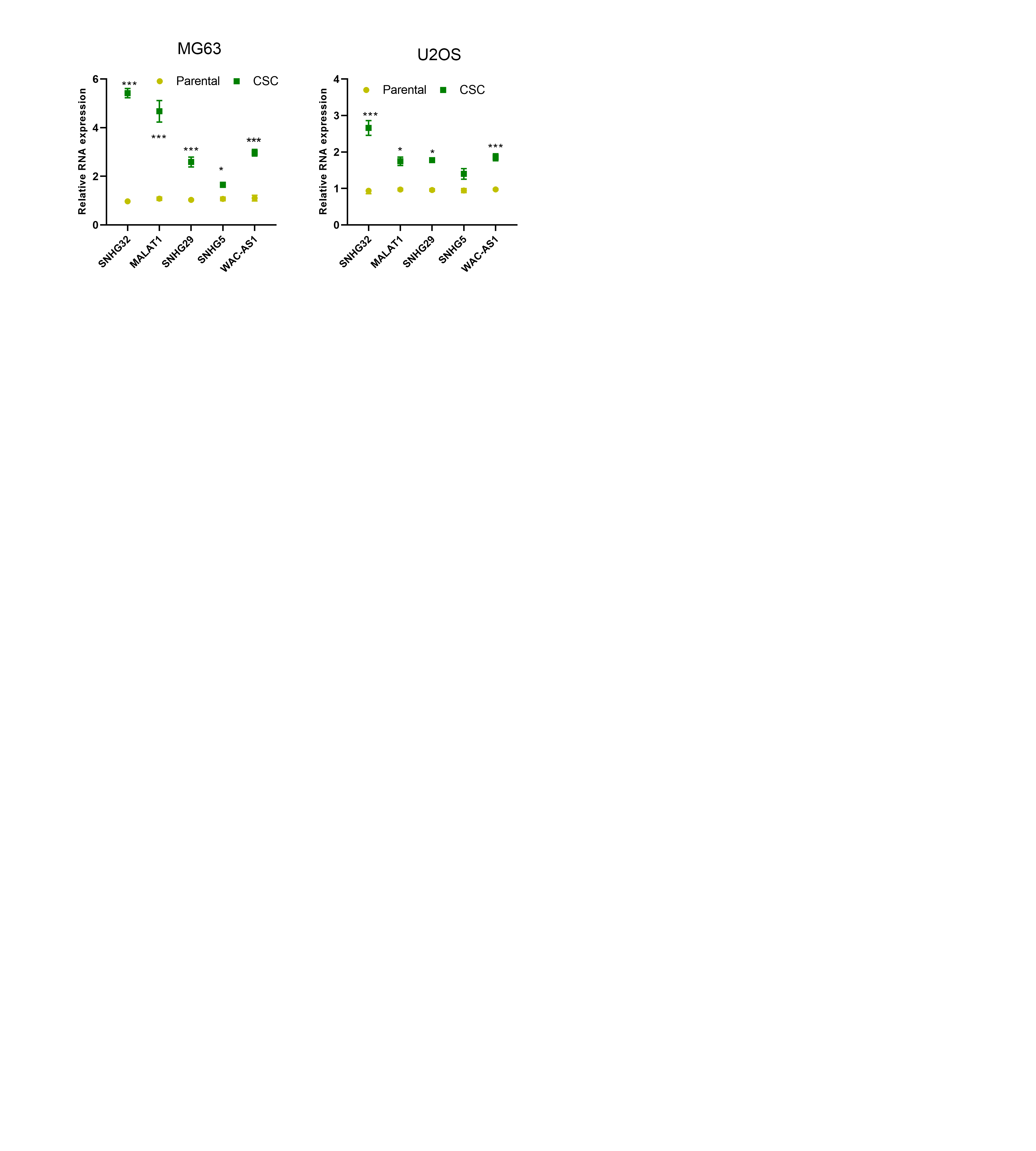

Supplement: Supplementary file 1 — Supplementary Material 1 [file 13062_2023_433_MOESM1_ESM.png]

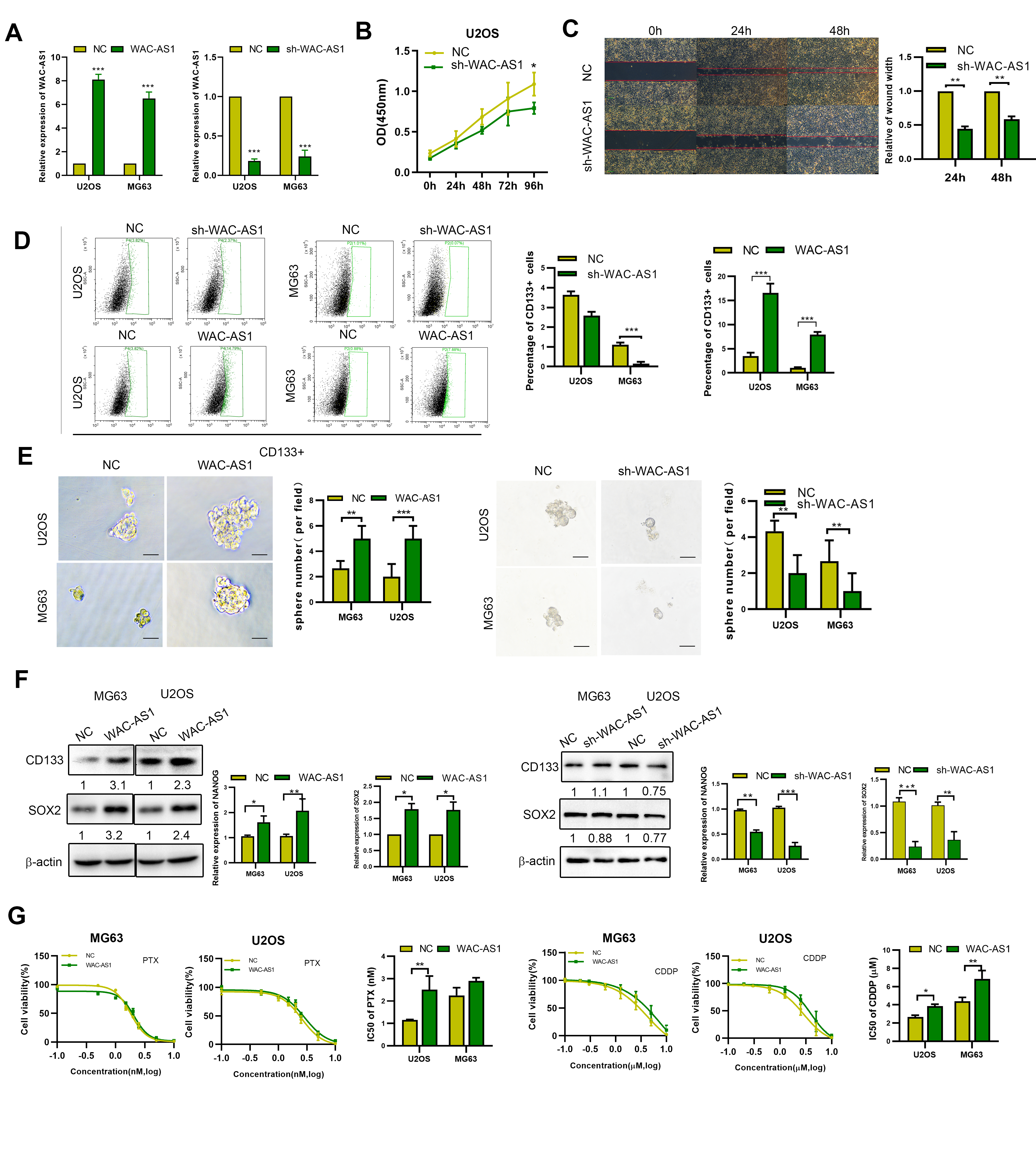

Supplement: Supplementary file 2 — Supplementary Material 2 [file 13062_2023_433_MOESM2_ESM.png]

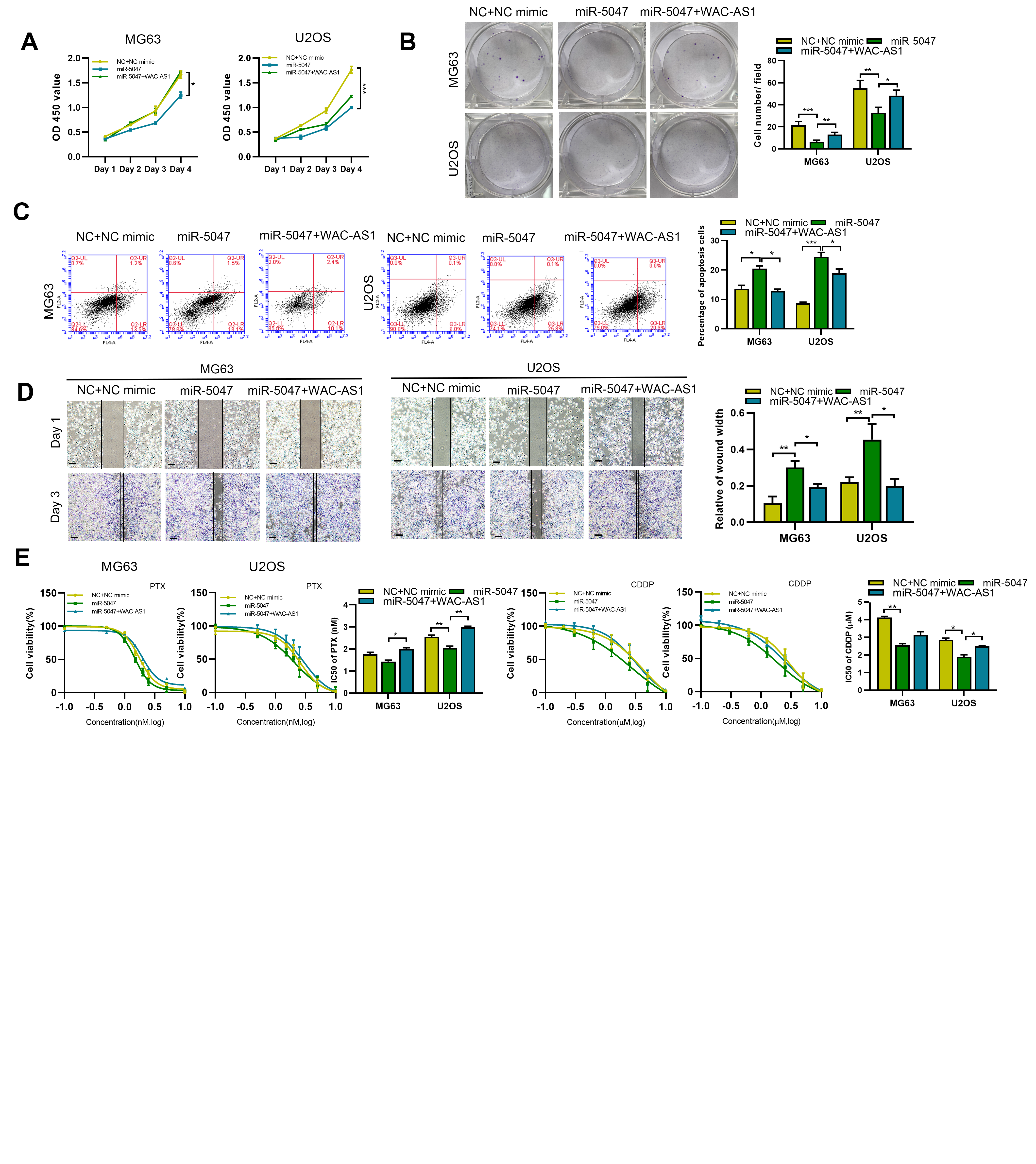

Supplement: Supplementary file 3 — Supplementary Material 3 [file 13062_2023_433_MOESM3_ESM.png]

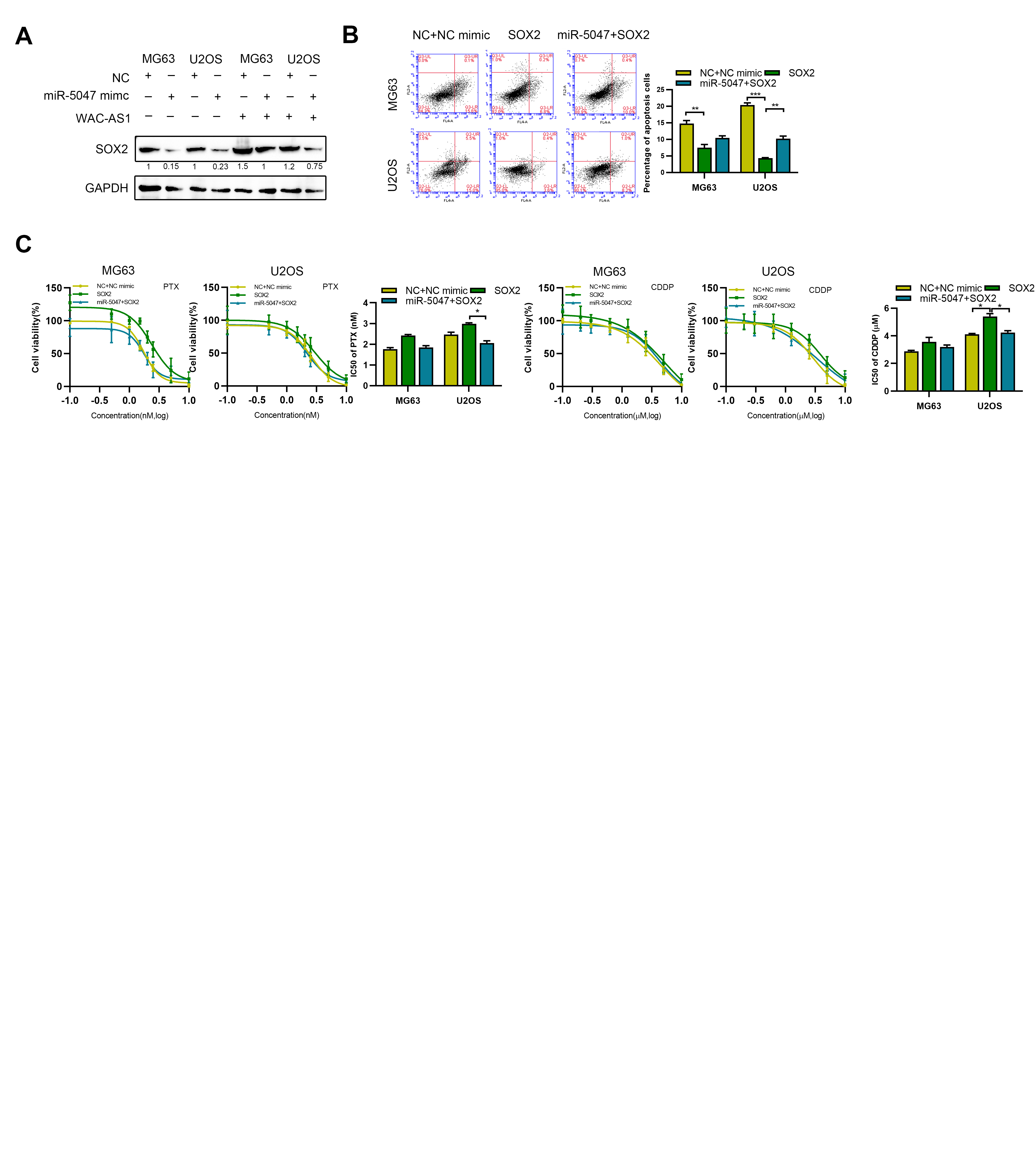

Supplement: Supplementary file 4 — Supplementary Material 4 [file 13062_2023_433_MOESM4_ESM.png]

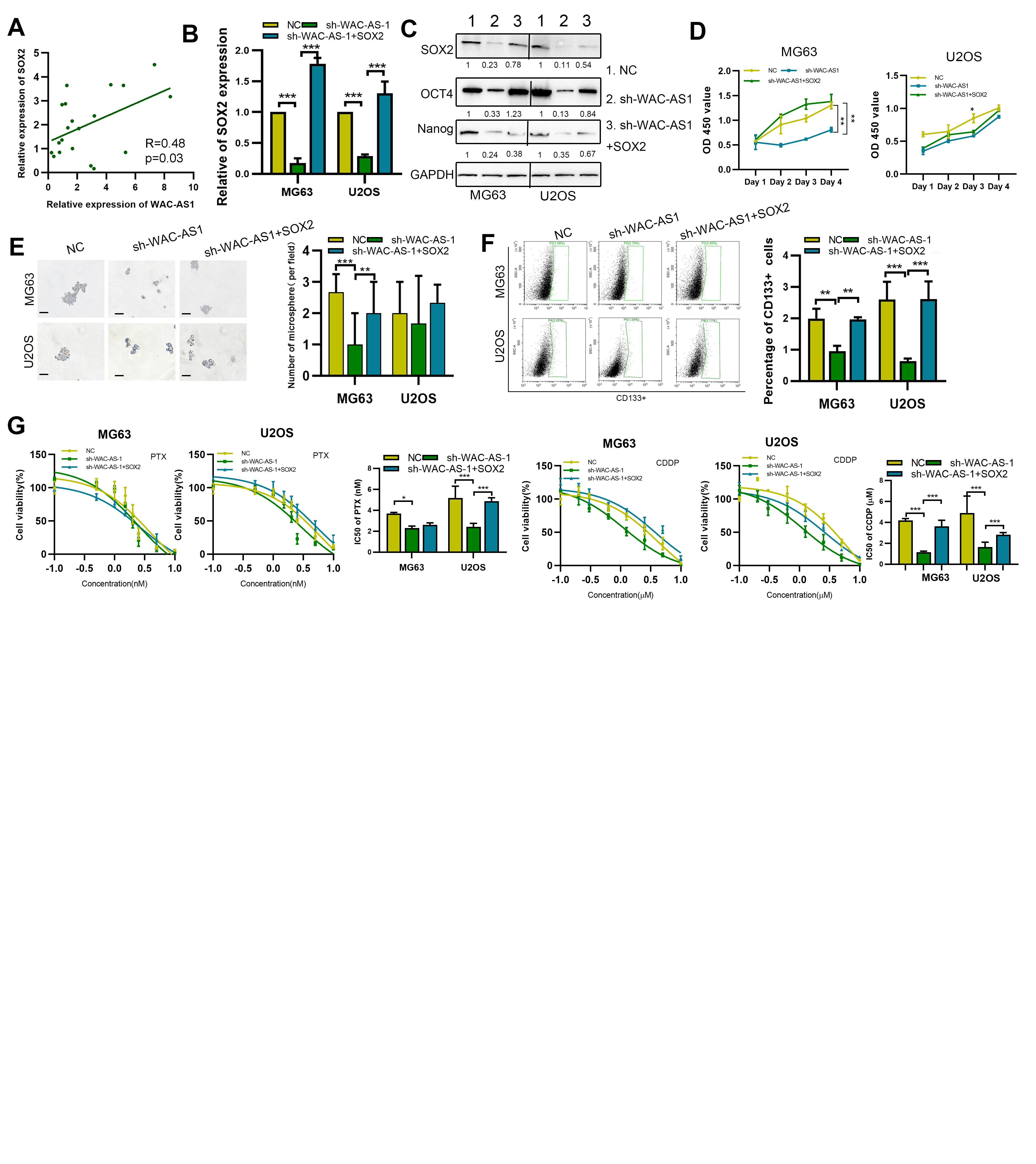

Supplement: Supplementary file 5 — Supplementary Material 5 [file 13062_2023_433_MOESM5_ESM.png]
