# Supplementary material for: LncRNA WAC-AS1 promotes osteosarcoma Metastasis and stemness by sponging miR-5047 to upregulate SOX2
Source: Biol Direct. 2023 Nov 14;18:74. doi: 10.1186/s13062-023-00433-2 (PMC10644615; doi:10.1186/s13062-023-00433-2)
Supplement: Supplementary file 6 — Supplementary Material 6 [file 13062_2023_433_MOESM6_ESM.docx]

| **Genes** | **Forward primer** | **Reverse primer** |
| --- | --- | --- |
| miR-5047 | CGTTGCAGCTGCGGTTG | AGTGCAGGGTCCGAGGTATT |
| miR-1301-3p | TTGCAGCTGCCTGGGAGT | AGTGCAGGGTCCGAGGTATT |
| miR-5579-3p | CGCGCGTTAGCTTAAGGAGTAC | AGTGCAGGGTCCGAGGTATT |
| miR-5590-3p | CGCGCGAATAAAGTTCATGTA | AGTGCAGGGTCCGAGGTATT |
| miR-142-5p | GCGCGCATAAAGTAGAAAGC | AGTGCAGGGTCCGAGGTATT |
| miR-1271-5p | GCGCTTGGCACCTAGCAAG | AGTGCAGGGTCCGAGGTATT |
| WAC-AS1 | CATTCGGTGCTGCGTCAT | GGAGTCTCAGGGAGCCTTCTA |
| SOX2 | CCCACCTACAGCATGTCCTACTC | TGGAGTGGGAGGAAGAGGTAAC |
| NANOG | TTCCCTCCTCCATGGATCTG | TGTTTCTTGACTGGGACCTTGTC |
| U6 | CTCGCTTCGGCAGCACA | AACGCTTCACGAATTTGCGT |
| β-actin | TGGCACCCAGCACAATGAA | GGAGTCTCAGGGAGCCTTCTA |
| Primer R1 | AGCCTCGTAAGTCACCTATCAT | CTGGAGGGAGTTCGCTGATT |
| Primer R2 | TCCTCCCCAACCCGATCC | CCTCCTTATCTGGAGCTGGC |
| SNHG32 | GGAACTGACCTAGCTCGTGG | GCTGGGCTTCCAGAGTTCAT |
| SNHG5 | CACAGTGGAGCAGCTCTGAA | CTCGTGGCACTAGCCAGAAA |
| MALAT1 | TGGTGGGGTGGGTTTAGGTA | TTCCCCCAGCTTCCCAATTC |
| SNHG29 | TTCCCGTTGTTATGGAGGGC | GCTCCAATACTCAGCTGCCA |
