# Supplementary material for: LncRNA WAC-AS1 promotes osteosarcoma Metastasis and stemness by sponging miR-5047 to upregulate SOX2
Source: Biol Direct. 2023 Nov 14;18:74. doi: 10.1186/s13062-023-00433-2 (PMC10644615; doi:10.1186/s13062-023-00433-2)
Supplement: Supplementary file 7 — Supplementary Material 7 [file 13062_2023_433_MOESM7_ESM.docx]

| **Variables** | **Univariable analysis** | | |
| --- | --- | --- | --- |
|  | **HR** | **95%CI** | **P value** |
| **Age(>25 vs ≤25)** | 0.55 | 0.254-1.193 | 0.13 |
| **Gender（Male vs Female）** | 1.393 | 0.62-3.13 | 0.43 |
| **Location (tibia vs other)** | 0.64 | 0.128-2.2 | 0.46 |
| **Lung metastasis (No vs Yes)** | 0.42 | 0.158-1.115 | 0.082 |
| **Invasion(Yes vs No)** | 1.28 | 0.398-4.116 | 0.679 |
| **Lymph node metastasis(No vs Yes)** | 0.80 | 0.364-1.757 | 0.579 |
| **WAC-AS1 expression (Low vs High)** | 0.505 | 0.231-1.102 | 0.086 |

**Supplemental table 2. The multivariate analyses of overall survival in OS patients (n = 72)**
